# Supplementary figures and images for: Dysregulation of the NLRP3 Inflammasome and Promotion of Disease by IL-1β in a Murine Model of Sandhoff Disease
Source: Cells. 2025 Jan 1;14(1):35. doi: 10.3390/cells14010035 (PMC11720672; doi:10.3390/cells14010035)

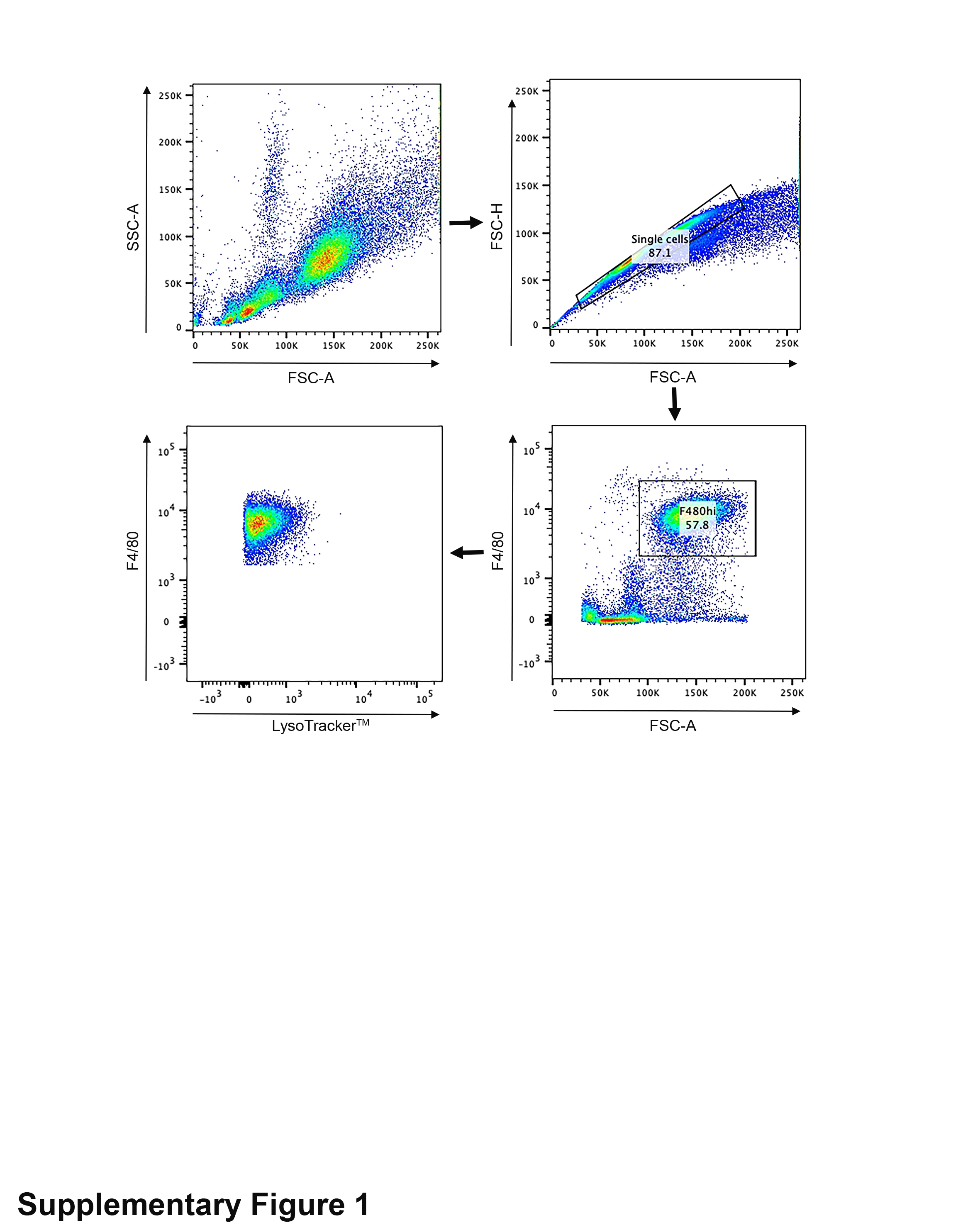

Supplement: Supplementary file 1 [file cells-14-00035-s001.zip › cells-3343944-supplementary1.pdf.tif]

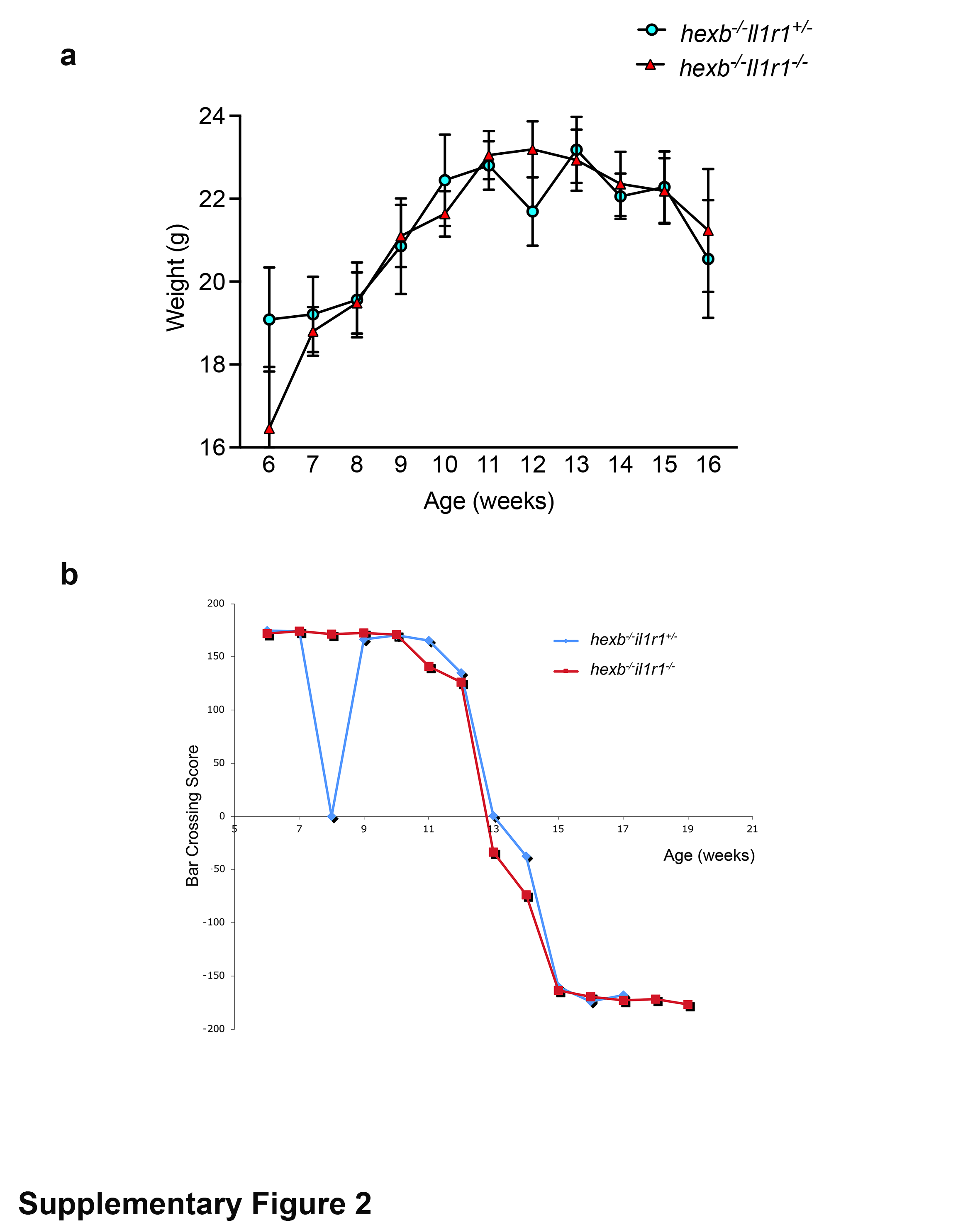

Supplement: Supplementary file 1 [file cells-14-00035-s001.zip › cells-3343944-supplementary2.pdf.tif]
